# Molecular Characterisation of Transport Mechanisms at the Developing Mouse Blood–CSF Interface: A Transcriptome Approach

**DOI:** 10.1371/journal.pone.0033554

**Published:** 2012-03-21

**Authors:** Shane A. Liddelow, Sally Temple, Kjeld Møllgård, Renate Gehwolf, Andrea Wagner, Hannelore Bauer, Hans-Christian Bauer, Timothy N. Phoenix, Katarzyna M. Dziegielewska, Norman R. Saunders

**Affiliations:** 1 Department of Pharmacology, The University of Melbourne, Melbourne, Victoria, Australia; 2 Neural Stem Cell Institute, Regenerative Research Foundation, Rensselaer, New York, United States of America; 3 University at Albany, Albany, New York, United States of America; 4 Department of Organismic Biology, University of Salzburg, Salzburg, Austria; 5 Institute of Cellular and Molecular Medicine, University of Copenhagen, Copenhagen, Denmark; 6 Department of Developmental Neurobiology, St. Jude Children's Research Hospital, Memphis, Tennessee, United States of America; Biological Research Centre of the Hungarian Academy of Sciences, Hungary

## Abstract

Exchange mechanisms across the blood–cerebrospinal fluid (CSF) barrier in the choroid plexuses within the cerebral ventricles control access of molecules to the central nervous system, especially in early development when the brain is poorly vascularised. However, little is known about their molecular or developmental characteristics. We examined the transcriptome of lateral ventricular choroid plexus in embryonic day 15 (E15) and adult mice. Numerous genes identified in the adult were expressed at similar levels at E15, indicating substantial plexus maturity early in development. Some genes coding for key functions (intercellular/tight junctions, influx/efflux transporters) changed expression during development and their expression patterns are discussed in the context of available physiological/permeability results in the developing brain. Three genes: *Secreted protein acidic and rich in cysteine* (*Sparc*), *Glycophorin A* (*Gypa*) and *C* (*Gypc*), were identified as those whose gene products are candidates to target plasma proteins to choroid plexus cells. These were investigated using quantitative- and single-cell-PCR on plexus epithelial cells that were albumin- or total plasma protein-immunopositive. Results showed a significant degree of concordance between plasma protein/albumin immunoreactivity and expression of the putative transporters. Immunohistochemistry identified SPARC and GYPA in choroid plexus epithelial cells in the embryo with a subcellular distribution that was consistent with transport of albumin from blood to cerebrospinal fluid. In adult plexus this pattern of immunostaining was absent. We propose a model of the cellular mechanism in which SPARC and GYPA, together with identified vesicle-associated membrane proteins (VAMPs) may act as receptors/transporters in developmentally regulated transfer of plasma proteins at the blood–CSF interface.

## Introduction

**Figure 1:**
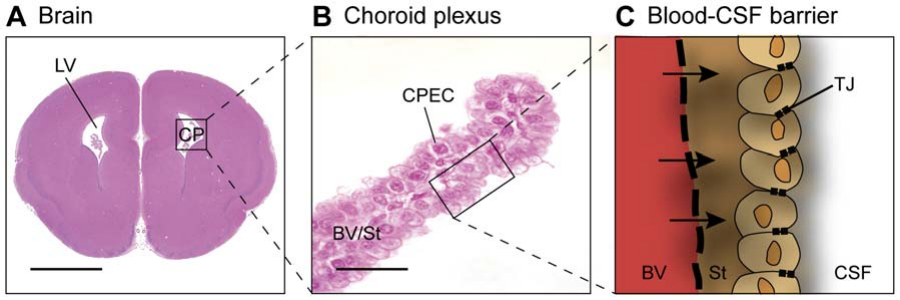
The choroid plexus and the blood–CSF barrier. **A.** The choroid plexuses are highly vascularised epithelial tissue that originates from the neuroependyma in the developing brain (ependyma in adult) and float freely in each of the brain ventricles (the two lateral, third and fourth). These specialised organs have two major functions: (i) to act as a diffusion barrier between the blood and the CSF; and (ii) they produce and secrete the CSF that fills the ventricles and subarachnoid spaces. **B.** The choroid plexuses are comprised of a central stroma with many blood vessels covered by a single layer of specialised epithelial cells, which rest on a thick basement membrane. **C.** The choroid plexuses are one of the circumventricular organs (i.e. they are positioned at sites around the margin of the ventricular system of the brain) and, like blood vessels in this region of the brain, the vessels of the choroid plexus stroma are fenestrated and the junctional strands linking adjacent endothelial cells are discontinuous. Accordingly, molecules are able to leave the blood vessels and enter the basement membrane of the plexus (arrows). However, a protective barrier is present in the choroid plexus, itself provided by tight, adherens, gap and other junctions between intimately apposed plexus epithelial cells – forming the blood–CSF barrier. The presence of many junctions between adjacent plexus epithelial cells from its first appearance in development means that the paracellular route for small molecules between blood and CSF is closed off in the embryo as well as in the adult. Abbreviations: BV, blood vessel; CP, choroid plexus; CPEC, choroid plexus epithelial cell; CSF, cerebrospinal fluid; LV, lateral ventricle; St, stroma; TJ, tight junction; Scale bar: 500 µm in A, 25 µm in B.

The central nervous system functions in a well-controlled environment and is protected by a set of mechanisms known collectively as the blood–brain barrier [1]. These mechanisms are present at five distinct interfaces: (i) the blood–brain barrier proper at the level of the endothelium of the cerebral blood vessels; (ii) the arachnoid barrier between the cerebrospinal fluid (CSF) in the subarachnoid space and the dura; (iii) the pia/glia limitans between the CSF in the subarachnoid space and extracellular fluid of the brain, which is much more complex in the embryo; (iv) the CSF-brain barrier, which is only a significant barrier in the embryo, created by separation of the ventricular system from the extracellular fluid of the brain by strap junctions in the neuroependyma and (v) the blood–CSF barrier at the level of the choroid plexus epithelial cells (**Fig. 1**). The importance of these barriers in normal neural development and in pathological conditions has been documented [2]–[5]. These same barrier mechanisms protect the brain from the effects of toxins and prevent the entry of many drugs [5], [6].

Most studies of brain barrier properties have concentrated on the cerebral endothelial cell interface (blood–brain barrier) with much less work having been done on the choroid plexus interface [7]. In the developing brain the differentiation and growth of the choroid plexuses occurs much earlier than most of the vascularisation of the brain. Thus it has been suggested that in early development the main portal of entry from blood into brain is via the choroid plexuses and CSF rather than via the sparsely distributed cerebral blood vessels [1], [8].

The main characteristics of each brain barrier interface include a morphological component in addition to cellular transport mechanisms. The key morphological feature is the tight junctions between the cells of the interfaces: cerebral endothelial cells at the blood–brain barrier and epithelial cells at the choroid plexus blood–CSF barrier [1], [9]. The cellular transport mechanisms encompass influx mechanisms for nutrients such as amino acids and vitamins, and efflux mechanisms, which are important barrier mechanisms that prevent the entry of many drugs and toxins into the brain [5], [10]. In addition, there are ion exchange mechanisms and water channels that define the key features of the brain's internal environment which are essential for normal neuronal function [8].

A key question for understanding brain development lies in determining to what extent these barrier mechanisms are functional in the embryonic and fetal brain and whether there are barrier mechanisms that are specific to the developing brain. In spite of numerous claims over nearly 100 years based on belief and poor experimentation that embryonic brain barriers are ‘immature’, it is now clear that fundamental barrier properties are present very early in development (see [1], [9]). Daneman and colleagues [2] have provided evidence of expression of numerous blood–brain barrier genes in the developing brain, but there is no equivalent information for the choroid plexuses.

An intriguing feature of CSF in the developing brain is its high concentration of protein—which has been identified in all mammalian species studied so far [11]–[15]. Experimental evidence showed that most of these proteins in CSF originate from blood plasma [15]–[18] although synthesis *in situ* in the choroid plexus also contributes [19]. Previous studies suggest that a specific recognition mechanism for individual proteins is present at the blood–CSF barrier, especially during early stages of brain development [16]–[18], [20], [21] but the molecular identity of this mechanism remains elusive. One molecule, secreted protein acidic and rich in cysteine (SPARC), has been proposed to be involved in targeting albumin to the blood-CSF interface—although it seems likely that SPARC is not the only transporter involved [22].

In this study we have used Affymetrix GeneChip arrays to describe the transcriptome of embryonic and adult mouse lateral ventricular choroid plexus and mined these datasets for intercellular junction and specific transporter genes. It is the first to describe the lateral ventricular choroid plexus transcriptome in the mouse embryo (at embryonic day 15) and to identify a set of genes whose expression is enriched compared with the adult. A transcriptome analysis of adult mouse choroid plexus has been published previously [23]. We report that several genes coding for proteins known to be albumin-targeting in other systems, are expressed within a subset of epithelial cells of the choroid plexus that are also immunopositive for albumin. We propose a way by which this mechanism for protein transfer across choroid plexus epithelial cells could operate.

## Materials and Methods

### Ethics statement

All animal experiments were conducted in accordance with the Public Health Safety Policy on the Humane Care and Use of Laboratory Animals (National Institutes of Health). All animal research protocols were reviewed and approved by the State University of New York – University at Albany Institutional Animal Care and Use Committee and registered with the US Office of Laboratory Animal Welfare (International Animal Care and Use Committee Registration A3621-01).

### Animal husbandry

Timed-pregnant and non-pregnant Swiss Webster female mice supplied by Taconic Farms Inc. (NY, USA) were used in this study. For general morphology and immunohistochemistry a range of embryonic (E) and postnatal (P) ages were used: E12, E13, E14, E15, E16, E19, P2, P15 and adult (10 weeks, 15–30 g). All embryos were staged according to the guidelines of Theiler [24]. For general histology paraffin-embedded brain sections from all ages were used (*n* = 6 animals at each age). For Affymetrix GeneChip arrays and qPCR validation lateral ventricular choroid plexuses from E15 (*n* = 100) and adult (*n* = 30) were used. For single-cell qPCR paraffin-embedded lateral ventricular choroid plexus sections from E15 and adult were investigated (*n* = 6 at each age). These ages were chosen as they correlate to the time when there is the maximum (E15) and minimum (adult) percentage of plasma protein-positive epithelial cells in the choroid plexus (see below and **Table S1**).

### Collection of brains for morphological studies

Embryonic animals were killed by decapitation, brains dissected out under cold 4% PHEM fixative (1.23 M paraformaldehyde, 1.29 M PIPES, 0.55 M HEPES, 0.20 mM EGTA, 0.16 M MgSO_4_) and post-fixed by immersion in Bouin's solution (Sigma, St Louis, MO, USA) for 24 hours. Postnatal animals were killed by cervical dislocation and fixed by perfusion with cold 4% PHEM fixative, for 10 minutes at 70% of cardiac output. Following perfusion, brains were dissected out and post-fixed by immersion in Bouin's solution for 24 hours. Once fixed, all brains were washed in 70% ethanol until clear, embedded in paraffin wax (Sigma) and 5 µm coronal sections cut.

### Collection of lateral ventricular choroid plexus

Animals were killed as above and brains dissected out under cold RNase-free phosphate buffered saline (PBS, pH 7.3). Cerebral hemispheres were separated and both lateral ventricular choroid plexuses removed and placed in fresh ice-cold RNase-free PBS. Plexuses were pooled from several litters, spun down, excess PBS removed, snap-frozen in liquid nitrogen and stored at −80°C. The choroid plexus consists of epithelium as well as blood vessels and mesenchymal stroma. However, the epithelium is the predominant cell type, suggested to represent up to 90% of the plexus tissue [25]. In this study lateral ventricular choroid plexus was taken *in toto*.

### Microarray screen of lateral ventricular choroid plexus

Total RNA was extracted from pools of E15 and adult lateral ventricular choroid plexus (from a minimum of 10 individuals in each pool) using the RNeasy Mini Kit, Qiashredder columns and gDNA removal columns according to standard supplier protocol (Qiagen, Valencia, CA, USA). Approximately 2 µg of total RNA from each of the six independent samples (three E15, three adult) were processed and hybridised to a GeneChip Mouse Exon 1.0 ST Array (Affymetrix, Santa Clara, CA, USA) using standard procedures (www.affymetrix.com). Microarray hybridisation and gel capture was performed by the Microarray Core Facility at the University at Albany Centre for Functional Genomics (Rensselaer, NY, USA). Array experiments were conducted according to the Minimum Information About a Microarray Experiment (MIAME) guidelines [26]. A checklist of these guidelines is provided in **Table S2**. An excel spreadsheet of the full comparison dataset is provided as **Table S3** and the array data have been deposited into the Gene Expression Omnibus (GEO, http://www.ncbi.nlm.nih.gov/geo/) with series accession number (GSE33009) and sample accession numbers (GSM818292, GSM 818293, GSM818294, GSM818295, GSM818926, GSM818927).

#### Affymetrix data analysis

For gene level analysis, the Affymetrix Power Tools software (Affymetrix) was used to normalise scanned images and to summarise the probe set. Briefly, raw array data was RMA normalised (robust multichip analysis, [27]) per chip median and filtered to include the top 80th percentile of genes expressed. Following an un-paired *t*-test (*p*<0.05), a Benjamini-Hochberg false discovery rate correction [28], [29] was applied before subjecting the list to a fold change cut-off of two. Further, more stringent discrimination of target lists was achieved using GoMiner™ software (Application Build 253; [30]). Targets were sorted using a combination of molecular and biological gene ontology terms.

### Real-Time qPCR validation

Total RNA was prepared from lateral ventricular choroid plexus as outlined above. First-strand cDNA was synthesised using a High Capacity RNA-cDNA conversion kit (Applied Biosystems, Carlsbad, CA, USA). Real-time PCR was performed using SYBR Green assay using the 7900HT Fast Real Time PCR System running SDS2.3 software (Applied Biosystems). Gene expression was determined relative to levels of expression of the transferrin receptor using the ΔΔCt method. Gene expression for transferrin receptor has been shown not to change during plexus development [31]. Standard deviations were calculated from triplicates of three separate samples in each instance. Primers for target genes were designed using the OligoPerfect™ Designer (Invitrogen, Carlsbad, CA, USA) and are listed in **Table 1**.

**Table 1:** Primers (5′→3′) used for qPCR validation of targets from array dataset.

Gene	Forward	Reverse
*Gypa*	GCATGGGTGAAAGCGTTAGT	GCCACAAAGCCTCTGAGTTC
*Gypc*	CCCCTCAGCTGTGCTATCTC	CCCTACAACATTCGGAGGAA
*Sparc*	GAGGGCCTGGATCTTCTTTC	CACGGTTTCCTCCTCCACTA
*Vamp1*	CTCCTCCCAACATGACCAGT	ACTACCACGATGATGGCACA
*Vamp5*	CCAAGACTTTAGCCCAGCAG	CTCGGAAGAAAGACGACCAG
*Vamp8*	CCTCCGAAACAAGACAGAGG	ACAGGGACTGAGCAGCACTT
*Tfrc*	TCGCTTATATTGGGCAGACC	CCATGTTTTGACCAATGCTG
*Abca2*	CATCTTGTGTGGCAACAACC	AAGCTTCGGATCTCTGTGGA
*Abca4*	AGGGAGAGCTGTGGTTCTCA	AGCAAGTCGTCCTTTGGAGA
*Abca5*	GGACAAATTCAGCCTTTCCA	GAAAGACTTGCGGCTACCAG
*Abca7*	GTTCTCTGGAGCTGGTTTCG	CAGGGCTGAGACTGTTGTCA
*Abcb2*	CAGAGAACCTGGGAGCAAAG	CACAACGCCACATAAACCAG
*Abcb3*	CCTGCCTTTCCTCATAGCTG	CCTGTCTTGGTCTCCTGGAA
*Abcb6*	CTGGCTGACATCATCATTGG	CGAAACTTGGCTCTCCACTC
*Abcb9*	GCGGTATTTTCACCCTCGTA	GCGGTTCTCATCAAAGAAGC
*Abcc1* (MRP)	CCCAGTGGGAACCTAGTGAA	CTGACTCCCAGCAAGGTCTC
*Abcg2* (BCRP)	TCACTGACCCTTCCATCCTC	AATCCGCAGGGTTGTTGTAG
*Abcg5*	GTCCTGCTGAGGCGAGTAAC	GCAGCATCTGCCACTTATGA
*Abcg8*	CCTCCGATTGCTTCTTTCAG	GGCAATCAGAGTCAACAGCA

### Laser capture and single cell PCR

Laser-capture microdissection was performed on paraffin-embedded tissue sections of E15 and adult mouse lateral ventricular choroid plexus. Randomly selected sections containing lateral ventricular choroid plexus were immunostained with antibodies to mouse albumin raised in goat (1∶5000, abcam, Cambridge, MA, USA, ab19194) or to mouse total plasma protein raised in rabbit (1∶5000, DAKO, Glostrup, Denmark, ZO177). For a list and specification of antibodies refer to **Table 2**. After washing with PBS+Tween20, sections were incubated with appropriate secondary antibodies conjugated to peroxidase (donkey anti-goat, 1∶1000, Millipore/Chemicon, Vienna, Austria, AP180P; goat anti-rabbit, 1∶1000, Sigma, A-9196). Final visualisation was completed with the 3,3′-diaminobenzidine reaction (DAB Kit, DAKO). Laser microdissection of immunopositive or immunonegative cells was performed with a Leica AS LMD microscope (Leica, Vienna, Austria) and captured cells placed in an RNase-free PCR cap. PCR was performed on single cells or on groups of 2–5 cells (negative controls). Extracted total RNA was directly transcribed into cDNA using the High Capacity RNA-to-cDNA master Mix (Applied Biosystems). PCR was performed using the smallest available intron-spanning TaqMan Gene Expression Assay (with amplicons <100 bp, Applied Biosystems) for *Sparc* (assay ID: Mm00470030_ml), *Glycophorin A* (*Gypa*, assay ID: Mm00494848_ml) and *Glycophorin C* (*Gypc*, assay ID: Mm00503602_ml). Amplification efficiency of the house-keeping gene *hypoxanthine-guanine phosphoribosyltransferase* (*Hprt*, assay ID: Mm01545399_ml) was used as an internal control. PCR products were separated on an agarose gel and stained with ethidium bromide.

**Table 2:** List and characterisation of antibodies.

Immunogen	ManufacturerSpecies/TypeCatalogue Number	Dilution/Application	Characterisation	Controls
Mouse albumin	abcamGoat/Polyclonal 1°ab19194	1∶1000/IHC	Stains expected band of *M_r_* 69×10^3^ on Western blot of whole mouse plasma.	Staining abolished after pre-incubation with purified mouse albumin.
Purified full length human Glycophorin A protein	abcamRabbit/Polyclonal 1°Ab35032	1∶10000/IHC	Stains expected band of *M_r_* 40×10^3^ on Western blot of whole mouse plasma and kidney tissue extract.	Staining abolished after pre-absorption with mouse plasma.
Recombinant full length human SPARC protein	abcamRabbit/Polyclonal 1°ab14174	1∶10000/IHC	-	Staining abolished after pre-absorption with human plasma.
Recombinant SPARC expressed in human embryonic kidney line HEK-293	Chemicon (Millipore)Rabbit/Polyclonal 1°AB1858	1∶100/IHC1∶2500/IHC1∶400/WB	Stains expected band of *M_r_* 34×10^3^ on Western blot of whole mouse plasma and kidney tissue extract (see **Fig. 4D**).	Staining abolished after pre-absorption with kidney tissue extract.
Purified immunoglobulin fraction of rabbit antiserum (total plasma protein)	DAKORabbit/polyclonal 1°Z0177	1∶2000/IHC	Reacts with mouse serum proteins (crossed immunoelectrophoresis) – at least 25 immunoprecipitates appear after staining with Coomassie Brilliant Blue (supplier bulletin).	Staining abolished after pre-incubation with mouse serum.
Immunoglobulins, mainly IgG, isolated from rabbit serum	DAKOSwine/polyclonal 2°Z0196	1∶200/IHC	-	-
Purified polyclonal IgG prepared from pooled mouse serum	DAKORabbit/polyclonal 2°Z0259	1∶200/IHC	-	-
	DAKORabbit/polyclonal 2°Z0228	1∶200/IHC	-	-

Abbreviations: 1°, primary antibody; 2°, secondary antibody; IHC, immunohistochemistry; WB, western blot.

### Immunocytochemistry

#### Total protein and albumin staining – cell counting

Selected 5 µm paraffin-embedded sections were dewaxed by heating to 60°C and immersion in histolene, rehydrated through graded alcohols and incubated with Peroxidase and Protein blocking agents (DAKO) for 2 hours each in a moist chamber at room temperature. After washing in PBS+Tween20, sections were incubated in an antibody against mouse serum raised in rabbit (1∶2000; DAKO, Z0177) or against mouse albumin raised in goat (1∶1000; abcam, ab19194) overnight at 4°C. The following day, sections were washed in PBS+Tween20 and incubated in appropriate secondary antibody (see **Table 2**) for 2 hours. A final incubation in species-specific peroxidase anti-peroxidase (1∶200) for 2 hours at room temperature was completed before antibodies were visualized with DAB (DAKO) for 5 minutes. The reaction was halted distilled water, before sections were dehydrated through graded alcohols and mounted with Ultramount #4 mounting medium (Fronine, Melbourne, VIC, Australia). Positive staining was recognised as a brown colour. Negative control sections included those where the primary antibody was omitted; these always appeared blank. Antibody dilutions were made in PBS-Tween20 buffer with 0.2% fish gelatine (Sigma).

Cell counting was performed on total plasma protein and albumin stained sections. Data are provided in **Table S1**.

#### SPARC and Glycophorin A immunocytochemistry

In some experiments sections were deparaffinised in xylene, rehydrated through graded alcohols, treated with a 0.5% solution of hydrogen peroxide in methanol for 15 minutes to quench endogenous peroxidase and then rinsed in TRIS buffered saline (TBS, 5 mM Tris-HCl, 146 mM NaCl, pH 7.6). Non-specific binding was inhibited by incubation for 30 minutes with blocking buffer (ChemMate antibody diluent S2022, DAKO) at room temperature. The sections were then incubated overnight at 4°C with one of two rabbit polyclonal antibodies against human SPARC (1∶10000; abcam, ab14174; or 1∶2500; Millipore/Chemicon, AB1858, see **Table 2**) or a rabbit polyclonal antibody against human GYPA (1∶10000, abcam, #35032). The two antibodies against SPARC gave similar appearances however the abcam antibody gave better definition of the reaction product. The sections were washed with TBS and incubated for 30 minutes with a peroxidase-labelled polymer conjugated to goat anti-rabbit/mouse immunoglobulins (EnVision™+ System/HRP K5007, DAKO). The sections were washed with TBS, followed by incubation for 6 minutes with DAB chromogen solution. Positive staining was recognized as a brown colour. The sections were counterstained with Mayer's haematoxylin and dehydrated in graded alcohols followed by xylene and coverslipped with DPX mounting media (Merck, Darmstadt, Germany). Antibody dilutions were made in blocking buffer (DAKO). Negative control sections were prepared by omitting the primary antibody and always appeared blank. Positive control sections were of mouse kidney (supplier technical bulletin and [32], [33] and were always positive.

### Western blot

To prepare samples for western blotting analysis, equal weights of plexus tissue were defrosted on ice and mixed with 20 µl PBS (with 0.2% Tween20) before disrupting with a glass mortar and pestle. Tissue was then centrifuged (3 minutes at 900 g, 4°C) to pellet excess cellular debris. Tissue homogenate (10 µl) was mixed with 2.5 µl sample buffer (0.5 M Tris-HCl, 0.4 M glycerol, 0.2 M β-mercaptoethanol, 10% w/v sodium dodecyl sulphate (SDS), 0.5% bromophenol blue, in distilled water). Samples were loaded on 10% Tris-HCl polyacrylamide gels (BioRad, Gladesville, NSW, Australia), with 4 µl of Kaleidoscope Molecular Weight Standard (BioRad). Gels underwent electrophoresis at constant voltage (100 V) in running buffer (25 mM Tris base, 192 mM glycine, 0.1% w/v SDS) for approximately 40 minutes, washed in cold Towbin buffer (25 mM Tris base, 192 mM glycine, 20% w/v methanol; [34]) and transferred at 200 V for 60 minutes to polyvinylidene difluoride membranes (BioRad). Membranes were blocked in a blocking solution of Towbin–Tween buffer (0.1 M Tris, 1.5 M NaCl, 0.1% Tween 20) with 50% low-fat soy milk overnight at 4°C. Membranes were probed using antibodies to mouse SPARC (1∶400, Millipore/Chemicon, AB1858) or antibodies to mouse GYPA (1∶500, abcam, ab20057), both raised in rabbit. Antibodies were diluted in blocking solution (see above). Incubation with primary antibody was for 2 hours at room temperature. Following washing in Towbin–Tween buffer, membranes were incubated with swine anti-rabbit immunoglobulins (1∶500, DAKO, Z0196) for 2 hours at room temperature. Membranes were washed and incubated for a further 2 hours with rabbit peroxidase anti-peroxidase (1∶500, DAKO, Z0113). After washing, specifically bound antibody was detected using DAB tablets dissolved with urea (DAB Plus kit, Sigma). The reaction was stopped in distilled water and membranes dried overnight before being photographed. Kidney was chosen as a control tissue as both SPARC and GYPA are known to be present in this organ [32], [35].

A list of all antibodies used in immunohistochemical, western blot and single-cell PCR methods is provided in **Table 2**.

### Statistical analysis

For statistical analysis InStat3 software (GraphPad, La Jolla, CA, USA) was used to perform Student's *t*-test and one-way ANOVA (Dunnett or Tukey post-hoc test) as appropriate.

## Results and Discussion

In order to identify different macromolecular transporters and changes in gene expression at the blood–CSF barrier in mouse development, Affymetrix GeneChip analysis was used to compare gene expression in choroid plexus collected from embryo versus adult mice. A previously described cell-counting method was used to ascertain the number of total and protein positive epithelial cells in the lateral ventricular choroid plexus of the mouse [18], [21], [36]. These data (provided in **Table S1**) were used to determine which age had the highest percentage of plasma protein positive choroid plexus epithelial cells to be compared with the adult in a microarray screen, thus E15 was chosen for comparison with adult. Lateral choroid plexus was chosen as this is the tissue on which most of the previous data on protein transfer are available [15], [16], [18], [21].

### Microarray results

**Figure 2:**
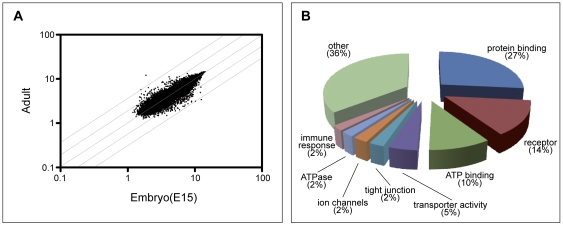
Gene expression in lateral ventricular choroid plexus of E15 and adult mice. **A.** The data are presented in a dot plot on a logarithmic scale, where each point represents a separate probe set on the GeneChip. Only probe sets that hybridized are represented, with no absent probe sets present in the plot. Data presented in plot are representative of a single GeneChip experiment, however three biological replicates were completed. Probe sets lying on the diagonal axis indicate similar levels of expression at E15 and adult. Validation using qPCR was completed on a select number of targets (see **Fig. 3** and **4**). **B.** Pie chart of the percentage of gene ontology of 1803 genes with altered expression in mouse lateral ventricular choroid plexus between the two ages. Protein binding, receptors and ATP binding genes accounted for 50% of all genes altered during development.

In total, 23239 probe sets displayed positive hybridization to the Affymetrix GeneChip. The relative gene expression in the plexus is graphically represented by a dot plot (**Fig. 2A**), where each dot represents a single probe set on the Affymetrix GeneChip, with the X and Y logarithmic axis representing the two ages used in the study. From this analysis the bulk of the genes expressed in the lateral ventricular choroid plexus lies near the diagonal axis, indicating that the expression levels do not differ between the two ages (i.e. fold change ≤2). Genes lying on or near this line were not included in further analysis, as the current study was focussed on describing those genes with altered expression in development. However, the large population of genes that did not significantly change their expression indicates a high degree of developmental maturity of the plexus even as early as E15 (three days after the lateral ventricular plexus first begins to develop). The number of probe sets that displayed changes in expression that were 2-fold or higher was 1805, of which 925 were enriched at E15 and 880 were enriched in the adult (see **Table S3**). The top 50 enriched genes are listed in **Table 3**. More stringent discrimination was applied to the gene list using GoMiner™ software (Application Build 253; [30]). Criteria were applied to identify gene targets according to their biological process, molecular function (transporter activity, receptor or vesicle-mediated transport, ion channel, ATPase, ATP-binding) or cellular component (tight junction, plasma membrane). We have previously suggested the presence of putative plasma protein-binding molecules at the blood–CSF barrier [17], [20], [22], therefore additional classification using molecular function (protein binding) was also carried out. Following classification into these broad biologically coherent categories, several classes of genes with altered expression in development were identified: cell adhesion (118 targets, **Table S4**), ion transport (101 targets, **Table S5**), solute carriers (56 targets, **Table S6**), efflux transporters (181, **Table S7**) and the largest group - protein binding (480 targets, **Table S8**, see also **Fig. 2B**). The relevant functional data sets are available as **Tables S4, S5, S6, S7, S8**. This report will concentrate on genes known to be associated with brain barrier function: tight junction and cell adhesion molecules, ion and water channels, efflux and influx transporters (especially potential protein transporters).

**Table 3:** Top 50 genes enriched in the embryonic (A) or adult (B) lateral ventricular choroid plexus of the mouse.

(A) Gene	GenBank ID	Fold Change	(B) Gene	GenBank ID	Fold Change
*H19*	BC025150	81.0	*Prlr*	BC096586	234.3
*Slc16a10*	BC052877	66.8	*Agxt2*	M22959	97.1
*Gpc3*	BC036126	60.4	*Cox8b*	BC086930	28.8
*Ddx3y*	BC021453	24.1	*Kl*	AB005141	27.4
*Rspo2*	NM_172815	19.3	*D12Ertd647e*	BC128276	25.3
*Hist1h1b*	NM_020034	17.6	*Scd1*	BC007474	24.1
*Ccna2*	BC052730	17.1	*Tgfb2*	BC011170	18.5
*Igf2bp1*	BC051679	16.6	*Hist1h2bc*	BC019673	18.3
*Racgap1*	AB030252	16.0	*Maob*	BC113788	17.9
*Sulf2*	AK129316	15.9	*Inmt*	BC013518	17.8
*Abcg5*	AF312713	14.1	*Cpxm2*	AF017639	16.4
*Ccdc67*	BC096547	13.1	*Npal2*	BC030399	15.5
*4930579J09Rik*	AY032666	12.8	*Sod3*	U38261	14.8
*Ppic*	M74227	12.1	*Sult1c2*	AY005469	14.5
*Slc6a15*	AY149280	11.4	*Ltc4s*	U27195	14.2
*Bex1*	BC058805	11.3	*Slc5a5*	AF235001	13.6
*Tpx2*	BC060619	11.2	*Tmem184a*	BC026659	13.3
*Fscn1*	BC052408	11.1	*Ppp2r2c*	BC059811	13.1
*Nrk*	AB020741	10.7	*Sytl2*	AB057754	12.3
*Rassf4*	BC060709	10.6	*F5*	U52925	11.7
*Prom1*	AF039663	10.6	*Sfrp5*	BC032921	11.7
*Tmem26*	BC117502	10.3	*BC055107*	BC055107	11.1
*Prc1*	BC059808	10.0	*Steap1*	BC061023	10.8
*Asb4*	BC046819	9.7	*Chdh*	BC039186	10.8
*Slc40a1*	AF231120	9.6	*Il1r1*	M20658	10.2
*Wfikkn*	AY308804	9.6	*Acsl6*	AY786360	10.2
*Rspo3*	AB055811	9.4	*Pla2g5*	AF162713	10.2
*Cdc2a*	U58633	9.2	*Ccl9*	U15209	10.2
*Gli3*	NM_008130	9.1	*Plek2*	AF170564	10.1
*Gdf5*	U08337	9.0	*Abca4*	AF000149	9.7
*Col3a1*	BC043089	8.8	*Rdh5*	AF033196	9.7
*Fn1*	BC051082	8.7	*Scx*	BC062161	9.6
*Plk1*	L19558	8.6	*Slc39a4*	BC023498	9.6
*Crabp2*	M35523	8.6	*Klf9*	Y14296	9.6
*Pbk*	BC020099	8.5	*Rrh*	BC046288	9.6
*Bub1b*	AF107296	8.4	*Igsf5*	BC004806	9.6
*Kif11*	BC060670	8.2	*Lrrtm2*	AY182027	9.4
*Kit*	BC052457	8.1	*Ppp1r1b*	BC011122	9.3
*Fstl1*	M91380	7.9	*Crhr2*	U19939	9.0
*A230106N23Rik*	BC116972	7.9	*Cd74*	BC096435	9.0
*Calcrl*	AB015595	7.8	*Nos1*	D14552	8.9
*Ccnb2*	BC008247	7.7	*Kcnh1*	U04294	8.9
*Sema6a*	BC059238	7.7	*Bdkrb1*	BC120684	8.8
*Pnck*	AF181984	7.7	*Car5b*	AF192978	8.7
*Ctnna2*	BC079648	7.6	*Gabrb1*	BC130258	8.6
*Col4a1*	J04694	7.5	*Slc41a2*	NM_177388	8.5
*Mfap2*	L23769	7.5	*Gpr37l1*	AB016602	8.5
*Cercam*	D16263	7.5	*Me3*	BC099935	8.4
*Casp7*	D16263	7.5	*Sult4a1*	BC054757	8.4
*Vcan*	BC115766	7.4	*Aox1*	AB017482	8.4

The top 50 most enriched lateral ventricular choroid plexus-specific gene targets in the embryo (**A**) or adult (**B**). Fold change column represents the increases in the number of transcripts for each gene compared to levels in opposing age (eg. a fold change of 3.0 for an embryonically enriched gene has expression 3-times higher than the adult). For full data set, refer to **Table S3**.

### Cell adhesion and tight junction genes

Tight junctions form the structural basis of the blood–brain and blood–CSF barriers [9], [37]. The physiological function of tight junction complexes in restricting paracellular diffusion between blood and the CSF is present very early in development [38], [39]. Key ultrastructural components such as the number and complexity of protein strands that make up the junction have been shown not to change significantly between very early gestation and adult [40]. Many of the typical proteins found in tight junctions such as ZO-1, occludin and claudins 1, 3–18 and 23 were present in the GeneChip array screen of mouse lateral ventricular choroid plexus. Their expression was similar at E15 and adult (**Table 4** and **Table S4**). *Cldn2* (Claudin 2) and several junctional transmembrane molecules, cytoplasmic adaptors and regulatory small GTPase transcripts did show an age-dependent enrichment in the lateral ventricular choroid plexus (**Table 4**). In particular, *Cldn2* was up-regulated 4-fold in the adult plexus. In contrast, in the embryo, the junctional adhesion molecule *Jam3*, proposed to be an integral part of tight junction complexes, displayed a 3-fold higher expression. The higher expression of *Jam3* in the developing plexus is important as this protein has been implicated in the establishment of the earliest cell-to-cell contacts that even precede tight junction formation [41]. The *Racgap1* (Rac GTPase activating protein 1), also important for the establishment of junctions, was enriched 16-fold at E15. Rac-1, in general, is a major regulator of barrier function and its activation is important for tight junction formation, which along with activation by Tiam1 controls tight junction biogenesis by binding to and activating the Par polarity complex [42]. Additionally, *Cdh2* (cadherin-2/N-cadherin) and *Cdh11* (cadherin-11), both up-regulated in the embryonic choroid plexus (**Table 4**) appear to be important in delineating compartments in the embryonic brain [43], [44] but have not previously been identified in the choroid plexus. *Pcdh18* (protocadherin-18, 6.8-fold increased expression in the embryo) is involved not only in cellular migration during development, but also in cell adhesion [45].

**Table 4:** Tight junction and associated proteins enriched in mouse lateral ventricular choroid plexus.

(A) Gene	GenBank ID	Fold Change	(B) Gene	GenBank ID	Fold Change
**Transmembrane**					
*Pcdh18*	BC052198	6.9	*Igsf5*	BC004806	9.6
*Cdh5*	BC054790	4.3	*Cldn2*	BC085494	4.3
*Cmtm3*	AY241870	4.0	*Marveld3*	BC025851	4.2
*Cdh2*	AB008811	3.4	*Cldn12*	BC024057	2.1
*Jam3*	BC024357	3.2			
*Cldn11*	BC021659	2.0			
**Cytoplasmic Adaptors**					
*Asb4*	BC046819	9.7	*Ankrd57*	NM_172939	4.9
*Ctnna2*	BC079648	7.6	*Ank*	DQ832285	3.3
*Lama4*	U59865	6.7	*Lin52*	BC048485	3.2
*Arhgap11*	AK129034	5.7	*Prkcz*	BC072590	3.0
*Dlg7*	AB076696	5.5			
*Cdh11*	BC046314	3.7			
**Regulatory Adaptors**					
*Racgap1*	AB030252	16.0			
*Rhobtb3*	AK129234	4.6			

List of proteins known to be associated with tight junctions that were up-regulated in either the embryo (**A**) or the adult (**B**) expressed as fold change compared to levels in other age. A full list of gene products involved in cell adhesion can be found in **Table S4**.

In the adult *Igsf5* (immunoglobulin superfamily 5/Jam4) was up-regulated nearly 10-fold (**Table 4**). The function of this adhesion molecule is dependent on simultaneous expression with other proteins such as Occludin, ZO-1 and Magi1 (membrane associated guanylate kinase with inverted domain structure-1) and were identified in the array screen but displayed no enrichment at either age. Occludin, Marveld2 (tricellulin) and Marveld3 are involved in stabilisation of tight junctions; transcripts for all 3 were detected, but only Marveld3 was differentially regulated (up 4.2-fold in the adult plexus). Although important for the stabilisation of tight junctions, lower expression of Marveld3 is reported not to disturb junction formation but does increase the trans-epithelial electrical resistance in cultures of epithelial cell lines [46], suggesting that Marveld3 may also be important for mediating paracellular ion permeability.

Genes coding for other intracellular accessory tight junctional proteins such as *Dlgh1/5*, *Mpp1/5/6/7*, and *Mpdz* were present in the embryo and showed no change in expression level compared to adult. These intracellular proteins are important components of the tight junction complex structure for two reasons: firstly they anchor the junction to the cytoskeleton; and secondly they may help to regulate the overall function of the structure [38]. Though these molecules are important for junction integrity they are not definitive for the barriers of the brain, but are more broadly components of tight junctions throughout the body [38]. The specific brain barrier accessory proteins *Pard3* and *Cingulin-like 1* (*jacop*) described by Daneman et al. [2] were unchanged in the lateral ventricular choroid plexus in development. This expression of these tight junction–specific molecules at E15 supports the previously described physiological and EM studies demonstrating functional maturity of the blood–CSF barrier even in very early developing brain [39].

The kinesin family member protein, *Kif11*, essential for moving vesicles and organelles within cells along microtubules, was increased 8-fold in the embryo compared to the adult, which could point to a greater importance of these intracellular transport mechanisms in the embryo. Other family members, *Kif2c* (important for removal of tubulin dimers from microtubules), *Kif20a*, *Kif22* and *Kif23* were all up-regulated in the embryo, 7.3-, 5.5-, 4.0- and 4.0-fold respectively. Both *Kif11* and *Kif2c* are highly ATP-dependent, in line with the highly metabolically active nature of these cells. These genes may be important in mechanisms that involve vesicular transport (see below).

### Ion transporters and channels

A key component of the internal environment of both the adult and developing brain is the stability of the ionic composition of the CSF, which is usually assumed to reflect that of brain interstitial fluid. The ionic composition of CSF in developing brain is different from the adult. Some CSF/plasma gradients are established very early in development, indicating the integrity of tight junctions is already restricting paracellular pathway diffusion of ions and that there are active ion pumps in the plexus epithelial cells (see [9] for summary). In the adult, the key mechanisms involved in CSF secretion are carbonic anhydrase, which generates HCO_3_^−^ that is transported across the epithelial cells and the sodium-potassium exchanger (Na+/K+ ATPase) in the apical membrane of the plexus epithelial cells [47]. In fetal rat choroid plexus the levels of carbonic anhydrase and Na+/K+ATPase are known to be much less than in the adult [8]. Similar results were obtained in the present study (**Table S3**). Thus the alpha subunit of the Na+/K+ATPase (*atp1a2*) was up-regulated in the adult over 7-fold: similarly, the carbonic anhydrases (*CA5b*, *CA8*, *CA13*) were up-regulated 4- to 9-fold in the adult (**Table S3**).

In the adult there was also enrichment in potassium ion channels (*Kcnh1*, *Kcnd3*, *Kcnd3*, *Kcnh2* and *Kcnk1*) ranging from 3 to 8-fold of levels seen at E15 (**Table 5**), while very few potassium-only ion channels showed increased expression in the embryo (though *Trpm5*, a potassium, sodium, caesium and calcium ion channel was 6.9 fold enriched, **Table 5**). The major extracellular cation, sodium, is transported by a number of solute carrier (*Slc*) gene products. Some were enriched in the embryo (*Slc6a13* – 4.6-fold, *Slc4a4* – 4.1-fold) and others in the adult (e.g. *Slc5a5* – 13.6-fold; *Slc24a4* – 7.8-fold, **Table 5** and **Table S5**). Anion transporters are also fundamental components for maintenance of the internal environment of the brain as well as CSF secretion, in particular Cl^−^ and HCO_3_^−^. Anion transporters known to be important in adult choroid plexus belong to the Slc4 family [46], [47]. Two that were up-regulated in the embryonic choroid plexus are *Slc4a1* (Cl^−^-HCO_3_ exchanger) and *Slc4a4* (Na^+^-HCO_3_^−^ co-transporter) see **Table 6**. No functional studies on individual ion transporters in developing brain or choroid plexus have been published, but it is possible to infer ion transport function from studies of CSF and plasma ion composition in the developing brain. Ion gradients between CSF and plasma are a characteristic of brain homeostasis [48]. Some ion gradients are established very early in brain development, for example Cl^−^ in fetal sheep [49] and neonatal rats [50]. This indicates that ion transport mechanisms are functional early in brain development, indicating functional activity of the ion transporters expressed in developing choroid plexus.

**Table 5:** Influx transporters and ion channels enriched in mouse lateral ventricular choroid plexus.

(A) Gene	GenBank ID	Fold Change	(B) Gene	GenBank ID	Fold Change
**Solute Carriers**					
*Slc16a10*	BC052877	66.8	*Slc5a5*	AF235001	13.6
*Slc6a15*	AY149280	11.4	*Slc39a4*	BC023498	9.6
*Slc40a1*	AF231120	9.6	*Slc41a2*	NM_177388	8.5
*Slc7a11*	AY766236	7.1	*Slc24a4*	AY156046	7.8
*Slc4a1*	BC053429	5.5	*Slc28a3*	BC013783	6.9
*Slc6a13*	BC029637	4.6	*Slc24a5*	AB085629	6.1
*Slc1a4*	BC043483	4.4	*Slc9a7*	BC058750	5.8
*Slc38a4*	AY027919	4.2	*Slc6a17*	AY155578	3.5
*Slc6a6*	L03292	4.1	*Slco1c1*	AY007379	5.2
*Slc4a4*	AF141934	4.1	*Slc4a10*	AK220501	5.0
*Slc7a1*	M26687	4.1	*Slc39a14*	AB177995	4.0
*Slc39a8*	BC006731	3.3	*Slc35f3*	BC115965	3.9
			*Slc13a4*	BC089161	3.9
			*Slc37a2*	AF121081	3.5
			*Slco1a5*	AF240694	3.4
			*Slc39a12*	BC089362	3.3
			*Slc46a1*	BC057976	3.2
			*Slc25a35*	BC019996	3.1
			*Slc22a5*	AF110417	3.0
**Ion Channels**					
*Racgap1*	AB030252	16.0	*Steap1*	BC061023	10.8
*Trpm5*	AB039952	6.9	*Kcnh1*	U04294	8.9
*Myb*	M12848	6.5	*Gabrb1*	BC130258	8.6
*Kcnip1*	AY171234	5.3	*Gabra4*	BC094603	7.9
*Gabrg*	BC099939	3.8	*Atp1a2*	BC036127	7.7
*Cngb3*	AJ243572	3.7	*Trpv4*	AF279672	7.6
*Ttyh3*	BC062917	3.6	*Chrnb4*	AF492840	6.9
*Gabra3*	M86568	3.4	*Nwd1*	BC024788	6.3
*Fxyd6*	BC051127	3.3	*Cacnb4*	BC026479	6.4
			*Slc9a7*	BC058750	5.8
			*Atp2a3*	BC026147	5.7
			*Atp13a4*	BC048410	5.6
			*Dmpk*	BC075715	5.4
			*Kcnd3*	AF107781	5.4
			*Trf*	BC092046	5.1
			*Pln*	BC061097	4.7
			*Best3*	AY450426	3.7
			*Kcnh2*	AF012868	3.5
			*Atp1b1*	BC094070	3.3
			*Kcnk1*	AF033017	3.2

Solute carriers and ion channels up-regulated in either the embryo (**A**) or the adult (**B**). In total, 157 targets were found for these gene ontology categories, with only those targets with fold change >3.0 shown above. A comprehensive list is presented in **Table S5, S6**.

**Table 6:** Expression and function of transporters in developing choroid plexus.

Transporter	Fold Change	Transport Function	Reference
*Slc16a10*	66.8	iodothyronines T3, T4	[92]
*Slc6a15*	11.4	neutral amino acids	[57]
* *Slc40a1*	9.6	iron	[93]
*Slc7a11*	7.1	cysteine, glutamate	[57]
*Slc4a1*	5.5	anion transporter, (Cl^−^-HCO_3_ exchange)	[46], [49]
*Slc6a13*	4.6	GABA transporter	[94]
*Slc1a4*	4.4	glutamate, neutral amino acids	[95]
*Slc38a4*	4.2	acidic & neutral amino acids	[57], [95]
*Slc6a6*	4.1	taurine	[57]
*Slc4a4*	4.1	Na^+^-HCO_3_^−^ cotransporter	[46]
*Slc7a1*	4.1	acidic amino acids	[57]
*Slc39a8*	3.3	zinc transporter	[96]

The expression levels for the transporters listed were obtained from Affymetrix array analysis of E15 and adult mouse choroid plexus. Only Slc4a4, Slc7a11 and Slc40a1 have previously been identified in choroid plexus. Fold change in expression compares E15 to adult choroid plexus. Superscript numbers indicate published studies showing transport into developing brain or CSF.

* Gene product ferroportin-1 identified in choroid plexus.

### Aquaporins

Aquaporins 1–9, 11, 12 were all expressed at similar levels in both embryonic and adult choroid plexus epithelial cells (**Table S2**). Only Aquaporin 1 has been established as having a clear function in water transport and CSF secretion in the choroid plexus [45]; it has previously been shown that this water channel is demonstrable immunohistochemically as soon as the choroid plexus differentiates and the expression level, as determined by real time RT-PCR did not change between E14 and adulthood [51].

### Amino acid influx transporters

Numerous members of the solute carrier gene family (*Slc*) of membrane transport proteins were present in the choroid plexus (**Table 5**, **Table 6** and **Table S6**). This large family (with over 300 members) is involved in the transport of many solutes across cellular membranes, and include ion channels and aquaporins (see above) facilitative transporters and active transporters. Three categories of expression of solute carriers were found in the developing mouse choroid plexus: those enriched in the embryo, those enriched in the adult, and those with relatively high expression levels that did not change with development. At E15, the mouse lateral ventricular choroid plexus contained many carriers involved in the transport of amino acids: *Slc16a10* (67-fold increase), *Scl7a11* (7-fold) and *Slc1a4*, *Slc38a4* and *Slc7a1* (all approximately 4-fold). These are summarised in **Table 6**. The transport of amino acids into the CSF and the developing brain is important for normal development. Most are directly involved in protein metabolism underlying cellular growth of the brain. Some are important because they act as carriers, for example, thyroid hormone transporters. The main ones so far described, *Slc16a2* (MCT8) and *Slco1c1* (Oatp14), have recently been identified in cerebral endothelial and choroid plexus epithelial cells of rat fetuses [52]. In the present study the expression levels of these transports were similar in E15 and adult choroid plexus, as were many other transporters in the MCT and Oatp families (**Table S3**). Another thyroid hormone transporter *Slc16a10* (TAT1, MCT10; [52]) showed strikingly high expression in embryonic choroid plexus (**Table 6**). However, *Slc16a10* has been previously reported as undetectable in the adult brain [53]. TAT1 (*Slc16a10*, MCT10) has also been proposed to be involved in endocytotic membrane sorting and recycling [54]; its expression in developing choroid plexus may be relevant to the protein transport mechanisms discussed below.

Direct measurement of amino acid transport in the brains of newborn animals has previously shown that many amino acids are indeed transported to a higher degree than in the adult [55]–[58]. This was supported by the finding in our study of enrichment of a large number of amino acid transporters at E15 (**Table 6**). In contrast, in the adult only one solute carrier responsible for the movement of amino acids was up-regulated (*Slc6a17*), although many more were actually present, either in levels not differing from the embryo, or less than at E15. **Table 6** indicates the available information on function of these transporter mechanisms in the developing brain; it is not yet possible to link directly the expression and transport function of individual genes in the developing brain. This is partly because many of the genes have only been identified recently, but also because of redundancy, the current experimental evidence is not adequate to define the function of individual genes. These earlier results from studies of amino acid transport are consistent with our overall finding of a higher level of expression of a large number of *Slc* genes in the embryonic choroid plexus compared to the adult.

### Efflux transporters

**Figure 3:**
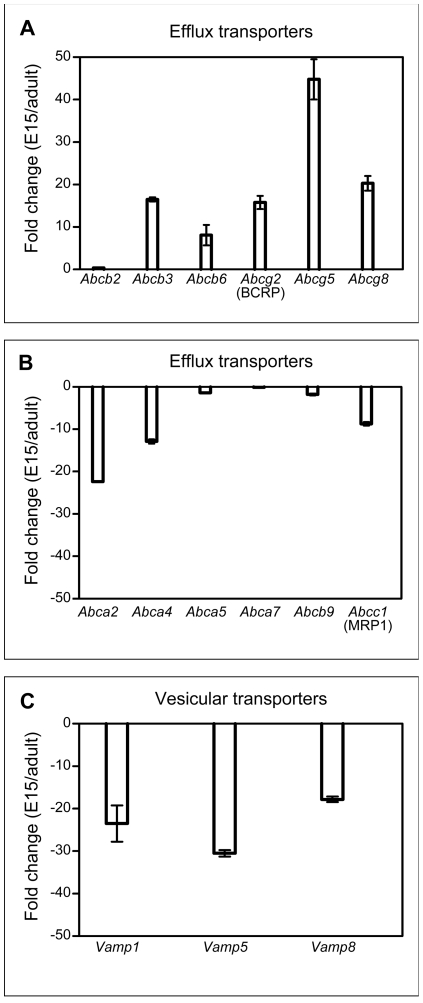
qPCR validation of microarray gene targets. Several members of the ABC family of efflux transporters were up-regulated in the embryo (**A**) and in the adult (**B**). The direction of these changes was in line with what was found using the Affymetrix GeneChip platform, though the magnitude of the expression was different. **C.** Three members of the vesicle-associated membrane protein (*Vamp*) family of proteins were down-regulated in the embryo (up-regulated in the adult).

The ATP-binding cassette (ABC) transporter family has been shown to be a key element of both the blood–brain and blood–CSF barriers from very early in development that can actively efflux a large number of lipophilic drugs [59]. ABC transporters are divided into seven distinct subfamilies: ABC1, multidrug resistance proteins (MDR/TAP), multidrug resistance-associated proteins (MRP), ALD, OABP, GCN20 and White. While many of these could be detected in the present screen of choroid plexus, most did not show evidence of developmental regulation (**Table 7** and **Table S7**). Two efflux transporters, *Abcg5* and *Abcg8*, were up-regulated 14.1- and 3.7-fold respectively at E15 compared to the adult. These have both been detected previously in adult rat choroid plexus, but only at a low level [58]. Several other ABC transporter genes have been shown to be differentially expressed in developing rat choroid plexus [10]. We found that BCRP (*Abcg2*) showed a strikingly higher expression in embryonic rat choroid plexus, whereas PGP (*Abcb1*) was not significantly different and MRP1 (*Abcc1*) and MRP4 (*Abcc4*) expression was significantly less in the embryo [10]. In the present study some of these genes were validated in mouse choroid plexus using RT-PCR (**Fig. 3**, **Table 6**). This confirmed that *Abcg2* (BCRP), Abcg5 and Abcg8 were substantially up-regulated in the embryonic choroid plexus as were Abcb3 and Abcb6 (**Fig. 3A**). In the adult *Abca2* and *Abca4* were up-regulated compared to E15 choroid plexus (**Fig. 3B**). The higher expression of BCRP (*Abcg2*) in the embryonic choroid plexus may be related to the physiologically hypoxic environment *in utero* [60] as it has been reported that hypoxia causes up-regulation of BCRP [61]. However, these latter findings need to be interpreted with caution, because of the *in vitro* conditions in which the experiments were conducted (see [6]).

**Table 7:** ABC transport/drug efflux gene expression in mouse lateral ventricular choroid plexus.

(A) Gene	Other IDs	array	qPCR	(B) Gene	Other IDs	array	qPCR
*Abcb3*	TAP2	-	16.5	*Abca2*		3.5	22.4
*Abcb6*	UMAT,MTABC3	2.6	8.1	*Abca4*	ABCR,RP19,RIM	9.7	12.9
*Abcg2*	BCRP	-	15.8	*Abca5*		2.5	1.4
*Abcg5*	Sterolin1,White3	14.1	44.8	*Abca7*	ABCX	4.5	-
*Abcg8*	Sterolin2,White4	3.4	20.3	*Abcb9*	TAPL	2.8	1.8
				*Abcc1*	MRP,MRP1	2.3	-

(C) Gene	Other IDs	array	(C) Gene	Other IDs	array
*Abca1*	TGD, CERP	NC	*Abcc3*	MRP3	NC
*Abca3*	ABC-C	NC	*Abcc5*	ARP5	NC
*Abca6*		NC	*Abcc6*	MRP6	NC
*Abca8a*		NC	*Abcc9*	SUR2	NC
*Abca8b*		NC	*Abcc10*	MRP7	NC
*Abca9*		NC	*Abcc12*		NC
*Abca12*		NC	*Abcd1*	ALD,ALDP	NC
*Abca13*		NC	*Abcd2*	ALDL1,ALDR	NC
*Abca14*		NC	*Abcd3*	PXMP1,PMP70	NC
*Abca15*		NC	*Abcd4*	PXMP1L,P70R	NC
*Abca16*		NC	*Abce1*	OABP	NC
*Abcb1a*	MRD1, P-GP	NC	*Abcf1*		NC
*Abcb2*	TAP1	NC	*Abcf2*		NC
*Abcb4*	MDR3	NC	*Abcf3*		NC
*Abcb8*	M-ABC1	NC	*Abcg1*	White	NC
*Abcb10*	M-ABC2,MTABC2	NC	*Abcg3*		NC
*Abcb11*	BSEP,SPGP,PGY4	NC	*Abcg4*	White4	NC
*Abcc2*	MRP2	NC			

Most enriched genes during development of the mouse lateral ventricular choroid plexus in the embryo (**A**) or adult (**B**). Some genes were present at both ages with no alteration in expression levels (**C**). Data obtained from Affymetrix GeneChip array and qPCR. Array targets were considered enriched with fold changes equal or greater than 2.0. See also **Table S7**. Abbreviations: NC, no change (in expression); -, not tested.

### Protein binding

The protein concentration in CSF in the developing brain is higher than in the adult (for review see [14]). It has been suggested that a protein-specific transfer mechanism may regulate movement of some proteins from blood into CSF and that this mechanism could be receptor-mediated [16], [17], [18], [20]–[22]. The physiological importance of high protein concentration in the developing CSF has been proposed to be: (i) maintaining high colloid-osmotic pressure gradients allowing normal brain development; and, (ii) act as a carrier for many essential molecules such as growth factors, hormones or nutrients (for review see [8]). The array dataset obtained in this study was mined for candidate genes with known protein-binding properties. The results are shown in **Table 8** **and Tables S2** and **S8**. Three of the targets identified, *Gypa*, *Gypc* and *Sparc* (osteonectin/BM-40/culture-shock protein) were selected for further study because of their reported role in the binding of albumin [62]. SPARC has also been recently reported in the choroid plexus epithelium [22].

**Table 8:** Protein binding targets enriched in mouse lateral ventricular choroid plexus.

(A) Gene	GenBank ID	Fold Change	(B) Gene	GenBank ID	Fold Change
*Hist1h1b*	NM_020034	17.6	*Kl*	AB005141	27.4
*Ccna2*	BC052730	17.1	*Tgfb2*	BC011170	18.5
*Bex1*	BC058805	11.3	*Cpxm2*	AF017639	16.4
*Fscn1*	BC052408	11.1	*Sod3*	U38261	14.8
*Rassf4*	BC060709	10.6	*Sytl2*	AB057754	12.3
*Wfikkn2*	AY308804	9.6	*Sfrp5*	BC032921	11.7
*Cdc2a*	U58633	9.2	*Il1r1*	M20658	10.2
*Gli3*	NR_027010	9.1	*Acsl6*	AY786360	10.2
*Gdf5*	U08337	9.0	*Igsf5*	BC004806	9.6
*Fn1*	BC051082	8.7	*Lrrtm2*	AY182027	9.4
*Plk1*	L19558	8.6	*Crhr2*	U19939	9.0
*Pbk*	BC020099	8.5	*Cd74*	BC096435	9.0
*Kit*	BC052457	8.1	*Nos1*	D14552	8.9
*Sema6a*	BC059238	7.7	*Gabrb1*	BC130258	8.6
*Ctnna2*	BC079648	7.6	*Atp1a2*	BC036127	7.7
*Cercam*	NM_207298	7.5	*Trpv4*	AF279672	7.6
*Cntn4*	BC115766	7.4	*Rnf152*	BC118961	7.5
*Nek2*	BC010302	7.3	*Dsg2*	AB072269	7.5
*Anxa2*	BC003327	7.1	*Bhlhb2*	BC010720	7.3
*Cdk6*	AF132483	7.0	*Cadm2*	NM_178721	6.9
*Pcdh18*	BC052198	6.9	*Hopx*	AF492703	6.6
*Uhrf1*	AF274046	6.8	*Cish*	BC022178	6.5
*Lama4*	U59865	6.7	*Zbtb20*	BC056446	6.5
*Myb*	M12848	6.5	*Cd9*	BC070474	6.3
*Dach1*	AF129510	6.2	*6330514A18Rik*	BC099679	6.2
*Col12a1*	U25652	6.1	*Lynx1*	BC037541	6.1
*Gypa*	BC148192	6.1	*Cacnb4*	BC026479	6.0
*Jarid1d*	AF127244	5.8	*H2-Aa*	BC031711	5.8
*Cd93*	AF081789	5.7	*Pcolce*	AB008548	5.8
*Nkd1*	AF343352	5.6	*Plp1*	M15442	5.8
*Trim59*	BC025430	5.6	*Tgfa*	U65016	5.7
*Slc4a1*	BC053429	5.5	*H2-D1*	U47325	5.7
*Cadm1*	AF434663	5.5	*Cltb*	BC070404	5.6
*Emid2*	BC075713	5.5	*A2m*	BC072642	5.6
*Sept11*	BC064466	5.2	*S100a1*	BC005590	5.4
*Plk4*	L29479	5.1	*Kcnd3*	AF107781	5.4
*Ezh2*	BC079538	5.1	*Tlr3*	BC099937	5.1
*Fkbp10*	BC029546	5.1	*Nfasc*	AJ543322	5.1
*Ccnb1*	BC011478	5.1	*Pcolce2*	BC051174	5.0
*Cited2*	BC057126	5.1			
*Dnajb13*	AF419292	5.1			
*Mfap4*	BC022666	5.1			

List of protein binding targets that were up-regulated in either the embryo (**A**) or the adult (**B**). In total, 479 targets were found for this gene ontology category, with only those targets with fold change >5.0 shown above. A comprehensive list is presented in **Table S8**.

Glycophorins are the main sialoglycoproteins of erythrocytes (for review see [63], [64]). Although there is evidence that many plasma proteins seem to be transferred from blood into the CSF, albumin has been most extensively studied [9], [15], [17], [20], [22]. GYPA and other albumin-binding proteins which are able to facilitate the transcytosis of native albumin in peripheral vascular beds and epithelial tissues have been reported to be virtually absent in adult brain capillaries [62]. Another albumin binding protein, SPARC (osteonectin/BM-40/culture-shock protein), is a soluble protein that can be found either incorporated into the plasma membrane of cells, floating free in the cytoplasm of cells or extracellularly in the blood plasma [65], [66]. SPARC is a glycoprotein that has been shown to be involved in protein binding (especially of albumin) in other epithelia [32], [35]. It has also been proposed to be a targeting molecule for albumin at the blood–CSF barrier [22] and to modulate cell matrix interactions, inhibit proliferation and affect growth factor signalling, as well as bind calcium and proteins such as albumin [66]–[71]. SPARC has been found localised to the basement membrane of many cell types [66]. Further analysis was restricted to targets related to protein transport properties of the choroid plexus.

**Figure 4:**
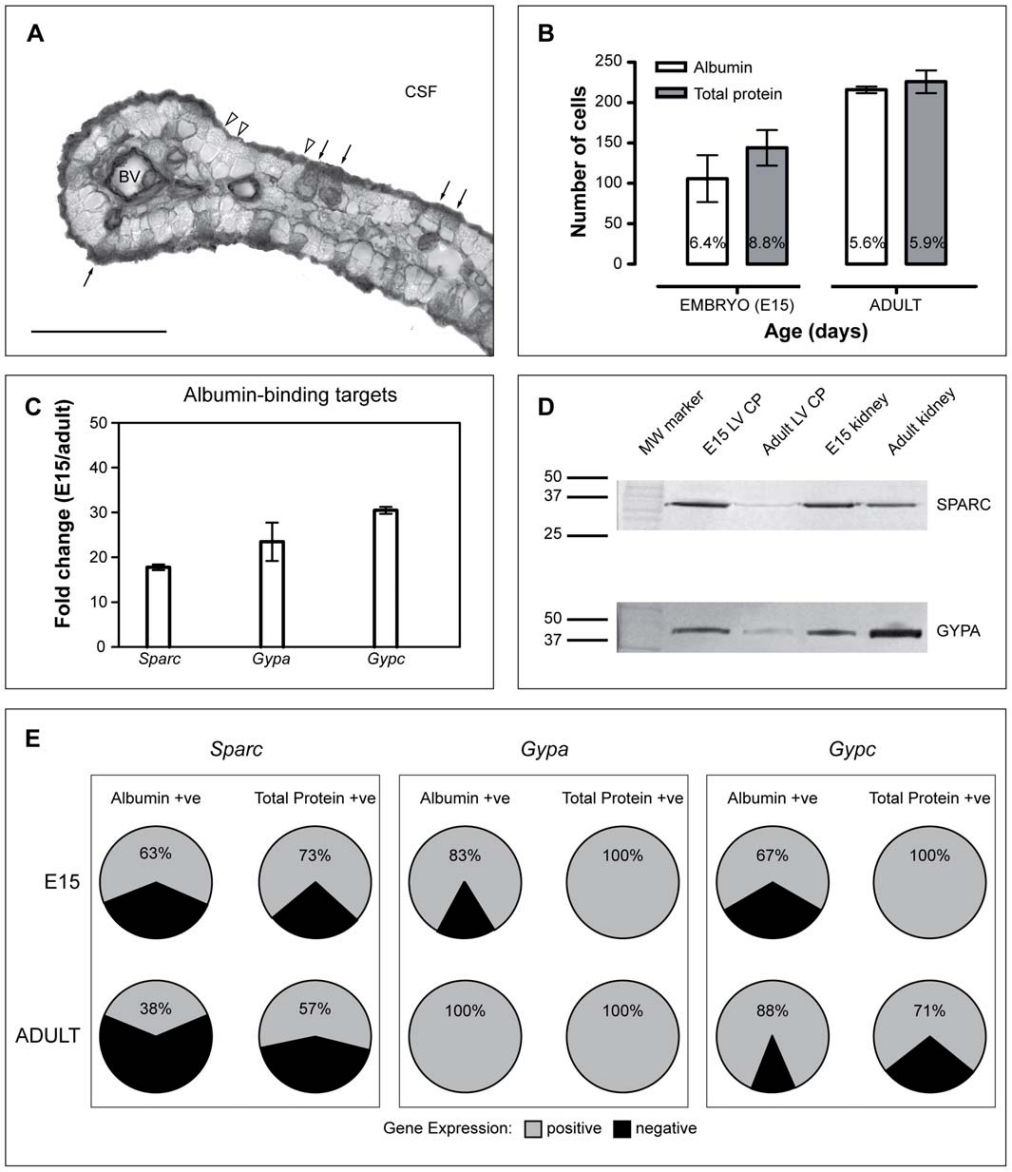
Co-localisation of albumin and total plasma protein with SPARC, Glycophorin A or C. **A.** Immunoreactivity in the lateral ventricular choroid plexus of E15 mouse embryo immunostained to detect endogenous albumin. Several epithelial cells of the plexus were deemed albumin-immunopositive (filled arrows), while all other cells were albumin-immunonegative (unfilled arrowheads). To be included in cell counts, the reaction product had to be present from basolateral to apical membranes and a cell nucleus had to be visible. **B.** Summary of cell count data from E15 and adult mouse lateral ventricular choroid plexus. The number of both total protein- and albumin-immunopositive plexus cells increased with age. As a percentage (numbers in bars), there was a decrease between E15 and adult, with E15 having the higher percentage of protein-positive cells of any age of the developing mouse. **C.** Quantitative PCR validation of microarray experiments showed that *Sparc*, along with *Gypa* and *Gypc* were up-regulated from about 20- to 30-fold in the embryo compared to the adult. **D.** Western blots of SPARC and GYPA in lateral ventricular choroid plexus and kidney (used as control) of E15 and adult mice. The level of SPARC did not change in kidney at the two ages: there was a substantial decrease in adult lateral ventricular choroid plexus compared to E15. A single dark staining band at the correct molecular weight (∼35 kDa, mouse SPARC MW 32 kDa) confirms specificity of the antibody. Levels of GYPA appeared to be higher in the adult kidney compared to E15. The highest level in the choroid plexus was in the embryo. A dark staining band at ∼40 kDa (mouse GYPA MW 43 kDa) confirms cross-reactivity of antibody. **E.** Single-cell PCR was performed on cells immunostained for either total protein or albumin. The percentage of immunopositive plexus cells that also displayed positive gene expression increased with age for both glycophorins, however it decreased for *Sparc*. Abbreviations: E, embryonic day; *Gypa*/GYPA, Glycophorin A; GYPC, Glycophorin C; LV CP, lateral ventricular choroid plexus; MW molecular weight (kDa).

Both *Gypa* and *Gypc* along with *Sparc* were found to be enriched in the lateral ventricular choroid plexus at E15 compared with the adult (6.1-, 3.7- and 2.2-fold respectively). These changes were confirmed with qPCR (**Fig. 4C**). Antibodies to SPARC and GYPA were used in western blots to estimate the change in levels of the protein present in developing choroid plexus cells. These results are presented in **Fig. 4D** and show that there were higher levels in the embryo than in the adult.

#### Single cell PCR

To ascertain the cellular co-localisation of albumin and its putative targeting proteins Sparc, Gypa and Gypc, laser dissection of both albumin- and total plasma protein-immunopositive plexus epithelial cells followed by single-cell RT-PCR was performed. The data are shown in **Fig. 4E**. For immunostained cells, 60–80% of albumin-immunopositive plexus epithelial cells showed expression of *Sparc* and both glycophorins at E15. For glycophorin targets this increased to 90–100% in the adult. In contrast, *Sparc*-expression in the adult was only present in approximately 40% of albumin-immunopositive plexus cells. Albumin- and total plasma protein-immunonegative cells of the plexus epithelium did not show amplification signals for either *Sparc* or the glycophorins at any age (**Fig. 4E**). In embryonic choroid plexus, numbers of cells immunopositive for total plasma protein that also showed high *Sparc* expression were higher than those immunopositive for albumin. All of the total plasma protein-immunopositive cells were also positive for both *Gypa* and *Gypc*. In the adult choroid plexus, the number of total plasma protein-immunopositive cells that were also *Sparc*-positive was less than in the embryo. All plasma protein immunostained cells were positive for *Gypa*, but for *Gypc* fewer plasma protein-immunopositive cells were positive than was the case for embryonic choroid plexus. The generally higher proportion of cells immunostained for total plasma protein than for albumin that also expressed these putative transporters indicates that SPARC and GYPA and GYPC are likely to be involved in transport of other plasma proteins, in addition to albumin, across choroid plexus epithelial cells.

#### Cellular & subcellular distribution of Glycophorin A

**Figure 5:**
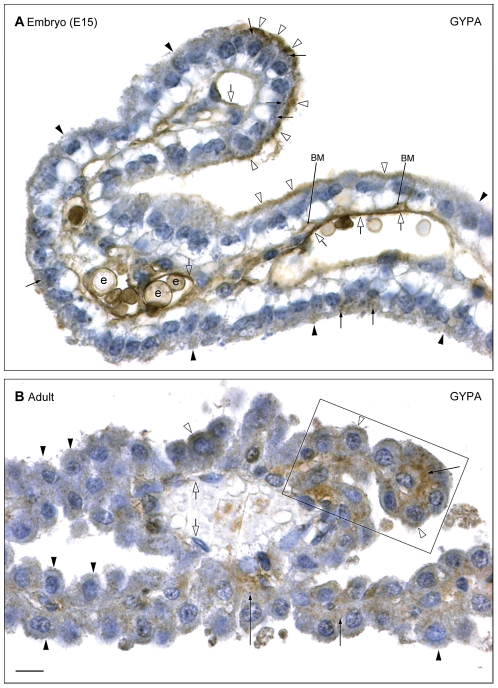
Cellular and subcellular distribution of GYPA immunoreactivity in E15 and adult choroid plexus. **A.** Embryonic day 15 (E15) lateral ventricular choroid plexus. The more medial younger segment of the plexus emerging from the hem and hippocampal anlage is shown in the lower and right side of the figure. This part of the plexus shows only weak immunopositive staining for GYPA (filled arrowheads). At the tip of the plexus most epithelial cells exhibit a strong granular immunoreactivity in the apical cytoplasm (arrows) and an apical membrane-associated surface immunostaining (open arrowheads). Basolateral cell membranes (BM) are also immunopositive, as are vascular endothelial cells, which show a marked immunoreactivity (open arrows), in particular in areas with basolateral and apical epithelial membrane-associated immunostaining. Strong membrane immunostaining of erythrocytes (e) is also evident. **B.** Adult lateral ventricular choroid plexus. A very weak fine granular cytoplasmic immunoreactivity is seen in almost all choroid plexus epithelial cells in contrast to a stronger immunoreactivity found in few small segments of the plexus (framed area). Note the apparent lack of cytoplasmic immunostaining in the apical-most part of most epithelial cells (filled arrowheads), but a distinct immunostaining of small vesicles within the cytoplasm (arrows). Very few cells exhibit an apical membrane-associated immunoreactivity (open arrowheads). There was no immunoreactivity for GYPA in vascular endothelial cell membranes (open arrows). Scale bar: 10 µm in all.

In order to investigate the possible role of one of these albumin-binding molecules in transport across the blood–CSF barrier, we have examined the distribution of GYPA at the cellular and subcellular level in lateral ventricular choroid plexus throughout mouse development from E12 onwards (see Methods) using a cross-reacting polyclonal antibody and the EnVision™+ system. Results are illustrated in **Fig. 5**. GYPA was detected in individual choroid plexus epithelial cells of animals at all ages from the first appearance of the lateral plexus at E13 (not shown), with a peak in intensity at E15–E16. Most epithelial cells exhibited weak immunoreactivity at this age but there were some cells, particularly in the older parts of the plexus (upper part of **Fig. 5A**), that showed strong immunoreactivity on the apical (CSF) surface (**Fig. 5A**). There was a marked decrease in immunoreactivity in the adult (**Fig. 5B**). The staining pattern was also different at different developmental ages. At E15 choroid plexus epithelial cells exhibited a predominantly peripheral plasma membrane-associated staining in both epithelial (apical and basolateral cell membrane) and endothelial cells (**Fig. 5A**). In contrast, in adult choroid plexus, immunoreactivity in general was absent from both apical and basolateral choroid plexus epithelial cell membranes, but was present intracellularly at a low level. In a few small segments of the plexus a stronger positive intracellular staining for GYPA was present (framed area in **Fig. 5B**).

At the subcellular level at the youngest ages, E13 and E14, a strong fine granular immunoreactivity for GYPA was present in the apical cytoplasm in the most mature choroid plexus epithelial cells opposite the hippocampal anlage. This segment of the plexus showed a weak staining of the epithelial basal membranes and of endothelial cell surfaces (not shown). The most intense staining was visible at E15 when pronounced membrane-associated GYPA immunoreactivity was visible along the apical surface of epithelial cells of larger segments of plexus (**Fig. 5A**). In the same areas basolateral membranes as well as endothelial cell membranes were also strongly stained (**Fig. 5A**). A few small vesicles in the apical cytoplasm showed reactivity whereas the nuclei were always negative (**Fig. 5A**). In the adult a marked decrease in the cell surface–associated epithelial membrane staining was observed (**Fig. 5B**). The cytoplasm exhibited an overall fine granular reactivity, with most immunoreactivity in the basal cytoplasm. A distinct immunostaining of small vesicles (arrows in **Fig. 5B**) was still present. Small segments of the plexus showed a stronger reactivity (framed area in **Fig. 5B**). There was no reactivity for GYPA in vascular endothelial cell membranes.

#### Cellular & subcellular distribution of SPARC

**Figure 6:**
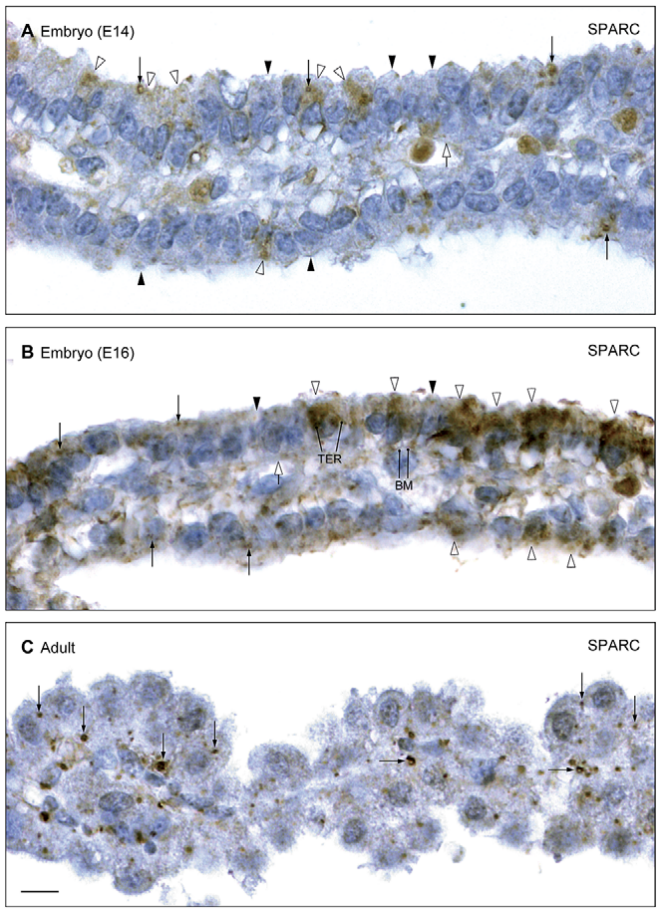
Cellular and subcellular distribution of SPARC immunoreactivity in E14, E16 and adult choroid plexus. **A.** Embryonic day 14 (E14) lateral ventricular choroid plexus. The most lateral (older) segment of the plexus close to the tip is shown in the upper part of the figure. In this area small subsets of SPARC-immunopositive epithelial cells exhibit a fine granular immunoreactivity (open arrowheads) and a few strongly immunostained large ‘vacuoles’ (arrows) in contrast to neighboring epithelial cells without immunoreactivity (closed arrowheads). Note the absence of SPARC-immunostaining in perivascular spaces and in endothelial cell membranes (open arrow). **B.** E16 lateral ventricular choroid plexus. A segment of the plexus similar to that shown in **A** exhibits many strongly immunoreacting choroid plexus epithelial cells (open arrowheads). The patchy reaction product in these cells is associated with a tubulocisternal endoplasmic reticulum (TER). Note the immunostaining along the basolateral cell membranes (BM) in the strongly SPARC-immunopositive cells. A few large granules (arrows) are present in the majority of the epithelial cells including those showing low SPARC-immunoreactivity (filled arrowheads). SPARC-immunostaining is absent from perivascular spaces and endothelial cell membranes (open arrow). **C.** Adult lateral ventricular choroid plexus. Note the apparent lack of cytoplasmic immunoreactivity in the apical-most part of the epithelial cells, but the pronounced immunostaining of large ‘vacuoles’ (arrows) which represent a combination of multivescicular bodies and lysosomes. There was no immunoreactivity for SPARC either in perivascular spaces or in individual endothelial cell membranes. Scale bar: 10 µm in all.

Cellular distribution of SPARC at the blood–CSF barrier was also investigated. Results are illustrated in **Fig. 6** **.** SPARC was detected in individual choroid plexus epithelial cells of animals at all ages, with a peak in intensity of immunoreactivity in E15–E16 embryos (Cf. **Fig. 6A and B**). As found for GYPA, most immunoreactivity for SPARC was observed in the apical cytoplasm in the most mature choroid plexus epithelial cells of the developing choroid plexus opposite the hippocampal anlage (**Fig. 6B**). Positive immunoreactivity was visible in E13 plexus epithelial cells (not shown), and following the peak at E15–16 there was a decrease in the strength of immunostaining by P2 (not shown) and adult (**Fig. 6C**).

At the subcellular level a positive fine granular staining was visible in small subsets of E14 choroid plexus epithelial cells throughout the cytoplasm with spared nuclei (**Fig. 6A**). Larger dense granules were also present. The perivascular space and endothelial cells of the choroid plexus showed no reactivity for SPARC. At E16 a more pronounced SPARC immunoreactivity was present in the most apical and central part of the part of epithelial cells in the positively-reacting segments of the plexus (**Fig. 6B**). The immunostaining reaction outlined the tubulocisternal system and extended along the lateral cell membrane to the basal membrane, which was also stained (**Fig. 6B**). There was no SPARC reactivity in endothelial cells forming the stromal centre of the plexus. In the adult there was an apparent lack of fine granular cytoplasmic reactivity in the apical-most part of the epithelial cells combined with an absence of tubulocisternal-associated cytoplasmic reactivity (**Fig. 6C**). However, the basal cytoplasm of most cells showed a strong staining reaction of large ‘vacuoles’, multivescicular bodies and lysosomes. There was no reactivity for SPARC either in perivascular spaces or in individual endothelial cell membranes.

### Vesicle-associated membrane proteins

The vesicle-associated membrane proteins (VAMP1, VAMP5 and VAMP8) are members of a family of SNARE proteins (soluble NSF attachment protein receptors), mostly involved in vesicle fusion [72]–[74]. There are seven members of this protein family in total: VAMP1/synaptobrevin, VAMP2, VAMP3/cellubrevin, VAMP4, VAMP5, VAMP7/Ti-vamp, and VAMP8/endobrevin [75]–[79]. These proteins are generally distributed in various post-Golgi structures, as well as on the plasma membrane of transporting cells. The intracellular traffic between different membrane compartments and across transporting cells involves a diverse range of membrane-enclosed intermediates, generated at plasma membranes via the action of several membrane proteins (such as the VAMP family) as well as cytosolic coat proteins [80]–[82].

**Figure 7:**
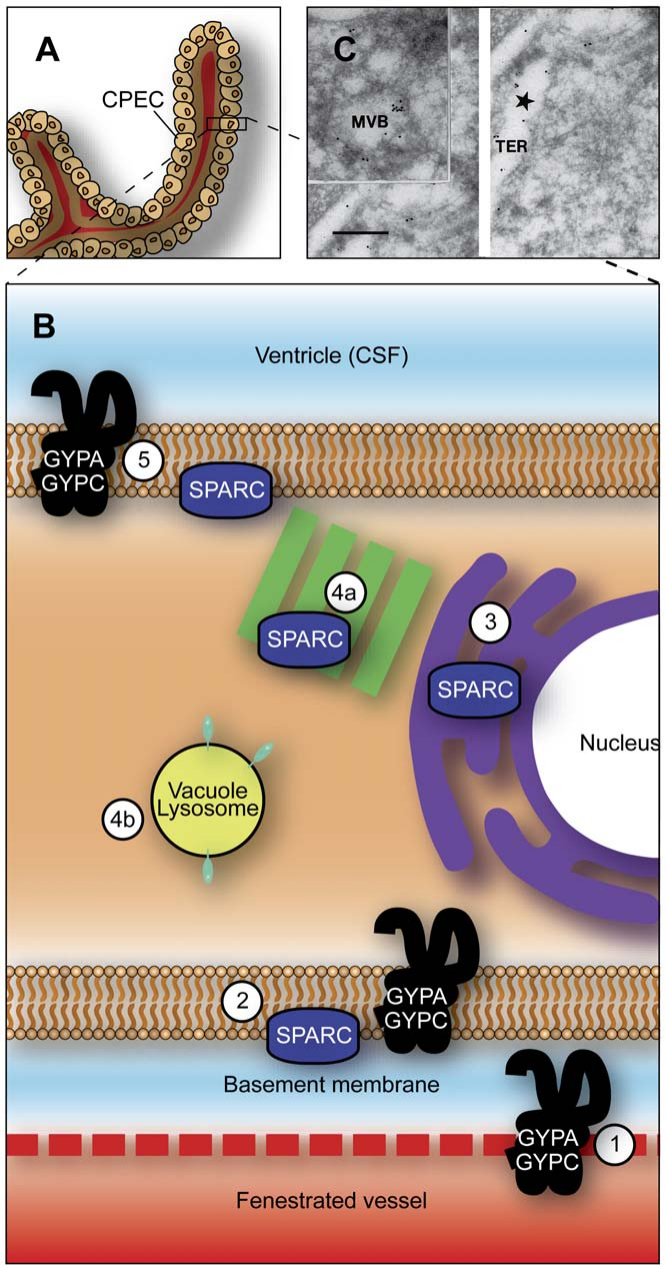
Proposed transepithelial pathway for albumin through choroid plexus epithelial cells. **A.** Whole choroid plexus showing single layer of epithelial cells sitting on thick basement membrane (see also **Fig. 1C**). **B.** Illustration depicting the suggested routes of albumin from plasma into CSF across the choroid plexus epithelium. GYPA/C in the endothelial cells may deliver albumin to the basement membrane (1) from where it can be taken up into the plexus epithelium by GYPA/C or SPARC (2). From here albumin may travel along a SPARC-specific pathway through the tubulocisternal endoplasmic reticulum (3, and see C) and Golgi (4a), or via a VAMP-mediated pathway in vacuoles, lysosomes or multivesicular bodies (4b, and see panel C). On the apical surface of the plexus epithelium, GYPA/C may be involved in efflux of protein from the cell into the CSF of the ventricles (5), as validated by extensive GYPA immunoreactivity in embryonic plexus (**Fig. 5A**). In the adult, the lack of immunoreactivity in the endoplasmic reticulum and Golgi (see **Fig. 5** and **Fig. 6**) along with increased expression of gene products for VAMP molecules (see **Fig. 3C**) suggest that the majority of transport possibly occurs via VAMP-mediated vesicular/lysosomal transport such as shown in (4b). **C.** Transmission electron micrograph of ultracryosection from E60 fetal sheep choroid plexus [87]. Immunolabelled human albumin 6 nm particles and sheep albumin 12 nm gold particles are shown to co-localise within the tubulocisternal endoplasmic reticulum. Abbreviations: CPEC, choroid plexus epithelial cell; CSF, cerebrospinal fluid; GYPA, glycophorin A; GYPC, glycophorin C; MVB, multivescicular body; TER, tubulocisternal endoplasmic reticulum. Scale bar: 0.2 µm in C.

Three *Vamp* targets were enriched in the adult lateral ventricular choroid plexus: *Vamp1* (2.3-fold), *Vamp5* (2.2-fold) and *Vamp8* (2.2-fold). The presence of the vesicle-associated membrane proteins in the epithelial cells of the lateral ventricular choroid plexus and their up-regulation in the adult supports the idea that there are two transfer mechanisms operating at the blood–CSF barrier: one specific for individual proteins; and another transfer mechanism, which is non-specific, occurring via vesicular uptake and release [18]. We confirmed GeneChip data using PCR (see **Fig. 3C**); and report enrichment of *Vamp1*, *5* and *8* in the adult mouse lateral ventricular choroid plexus. The expression of *Vamps* in plexus epithelial cells indicate that they may be involved in translocation of albumin and other plasma proteins across the choroid plexus epithelium, particularly in the adult. Thus VAMP5 is present on the outside of both the basolateral and apical membrane of the cell, helping to package molecules in both the blood and CSF and inside the cell, while VAMP1 is present on the cell membrane, working in tandem with Vamp5 [83], [84]. The third family member investigated in the current study, VAMP8 is probably present in cytoplasmic endosomes of the choroid plexus epithelial cell and is involved in removing unwanted material from the cell [85], [86]. Though these transcripts displayed up-regulated expression in the adult plexus, they were also present in the embryo. As mentioned above, many lysosomes were seen in the apical portion of the plexus epithelial cells that were immunopositive for the albumin-binding protein SPARC. It is possible that the SPARC-mediated recruitment of albumin into the epithelial cells of the choroid plexus on the basolateral (blood) surface is coupled with the VAMP-mediated uptake of albumin, into vesicles with exocytosis of albumin on the apical (CSF) surface into the CSF via an endosomal/lysosomal system within the cytoplasm of the plexus epithelial cells (4b in Figure 7).

### Proposed mechanism for plasma protein transport across choroid plexus epithelial cells

The present results provide additional evidence for a protein-specific transfer mechanism that contributes to regulation of the internal environment of the developing brain. Previous evidence suggested that this mechanism is particularly important in early development [16]–[18], [20], [21]. This is supported by the present finding that putative albumin receptors, SPARC, GYPA and GYPC are up-regulated in the embryonic choroid plexuses of mice, whereas expression of several members of the VAMP family of proteins is higher in the adult. These findings support the previous suggestion that two protein transfer mechanisms operate in parallel at the level of the choroid plexus epithelial cells of the blood-CSF barrier: one specific for individual plasma proteins in the blood to CSF direction, operating predominantly during early development; and a second, non-specific vesicular uptake mechanism in both blood to CSF and CSF to blood directions, operating both during early development and in the adult. The specificity of the protein specific transfer mechanism appears to be due to the presence of protein receptors on the basolateral surface of the choroid plexus epithelial cells. However, a direct link between these putative albumin transporters and albumin itself still needs to be established. These studies are in progress.

Some of the differences in subcellular distribution of SPARC and GYPA demonstrated by immunocytochemistry suggest that they may play different but complementary roles in albumin recognition and transport from blood to CSF in epithelial cells of the developing choroid plexus. A proposed mechanism for this transfer is illustrated in **Fig. 7**. Thus GYPA in the endothelial cells of vessels in the choroid plexus stroma may deliver albumin to the interstitial space from where it is taken up into the epithelial cell via the GYPA on the basolateral surface of the plexus cells. Immunoreactivity for SPARC in the tubulocisternal endoplasmic reticulum pathway (refer to **Fig. 6B**; [87], [88]; see also **Fig. 7C**) early in embryogenesis suggests the involvement of SPARC in transcellular transport of albumin [86]. SPARC-immunopositivity of the Golgi apparatus confirms that this glycoprotein is synthesised by the plexus epithelial cells as also shown by the positive single cell PCR and immunocytochemistry results. In contrast, in the adult there was a lack of fine granular cytoplasmic reactivity in the apical-most part of the epithelial cells combined with an absence of tubulocisternal-associated cytoplasmic reactivity (**Fig. 6C**). However, the basal cytoplasm of most cells showed a strong staining reaction of large ‘vacuoles’, multivescicular bodies and lysosomes, perhaps correlating with the up-regulated VAMPs 1, 5 and 8. A summary of the steps in this proposed pathway is provided in **Fig. 7**.

### Conclusions

In this study we provide a searchable resource for the elucidation of gene expression at the blood-CSF barrier in the embryonic mouse compared to the adult. This is the first gene profiling dataset available for the study of choroid plexus epithelial cells during development and we have reported a comprehensive list of transcripts enriched in the blood-CSF barrier of both the embryo and the adult. These targets encode those involved in metabolic activities, junction formation, influx and efflux transporters, protein binding and receptors.

One outcome is that it is now possible to make some comparisons between expression of specific genes in the embryonic choroid plexus and published functional data for transporters, tight junction permeability, protein binding and receptors. The findings also help to explain number of important previous findings. For example it was reported many years ago that glutamate is toxic to the brain if administered in the neonatal period [89]. Some attributed this to “immaturity” of the blood-brain barrier [90]. However, it can now be seen that the barrier contribution to toxicity is much more likely to be due to greater transport by e.g. Slc1a4 (**Table 6**). Another example is the recent report of serotonin synthesis by the placenta that appears to be important for forebrain development [91]. Numerous serotonin receptors (HTR1-7) were found to be expressed in embryonic choroid plexus (**Table S3**) suggesting this as the site of entry of serotonin into the developing forebrain. The finding of several albumin binding proteins in the route between blood and CSF across choroid plexus epithelial cells reinforces earlier evidence for a specific protein transport mechanism in the developing choroid plexus. Thus the mechanisms responsible for the nature and control of the internal environment of the developing brain, supply of nutrients and exclusion of toxins appear to be well established in the embryonic choroid plexus

## Supporting Information

Table S1 **MIAME Compliance Checklist.** MIAME describes the Minimum Information About a Microarray Experiment that is needed to enable the interpretation of the results of the experiment unambiguously and potentially to reproduce the results [26].
(DOCX)

Table S2 **Complete data set.** The spreadsheet contains a comprehensive list of probe sets from the GeneChip Mouse Exon 1.0 ST Array, with raw (normalised) data for E15 and adult mouse, fold change between embryo and adult (with a fold change cut-off of 2) and Gene Ontology classifications.
(DOCX)

Table S3 **Total and plasma protein positive choroid plexus epithelial cells during normal mouse development.** Data are expressed as mean ± s.e.m. rounded to the nearest whole cell. These values are for cells actually counted and represent approximately 10% of all choroid plexus epithelial cells at each age. % Total cells is the percentage of protein positive cells in relation to total plexus cell numbers. The lateral ventricular choroid plexus was not present in E12 embryos, hence no cell count data is available at this age. These data were used to select ages for microarray screening – the highest percentage of protein positive cells was seen at E15, and this age was compared with adult for the remainder of the study. *n* refers to the number of animals. *comparing youngest and oldest ages. Abbreviations: E, embryonic day; P, postnatal days.
(XLSX)

Table S4 **Cell adhesion specific genes.** A dataset containing probe sets from the GeneChip Mouse Exon 1.0 ST Array, with raw (normalised) data for E15 and adult mouse, fold change between embryo and adult (with a fold change cut-off of 2) and Gene Ontology classifications.
(XLSX)

Table S5 **Ion channel specific genes.** A dataset containing probe sets from the GeneChip Mouse Exon 1.0 ST Array, with raw (normalised) data for E15 and adult mouse, fold change between embryo and adult (with a fold change cut-off of 2) and Gene Ontology classifications.
(XLSX)

Table S6 **Transporter activity specific genes.** A dataset containing probe sets from the GeneChip Mouse Exon 1.0 ST Array, with raw (normalised) data for E15 and adult mouse, fold change between embryo and adult (with a fold change cut-off of 2) and Gene Ontology classifications.
(XLSX)

Table S7 **Efflux transporter specific genes.** A dataset containing probe sets from the GeneChip Mouse Exon 1.0 ST Array, with raw (normalised) data for E15 and adult mouse, fold change between embryo and adult (with a fold change cut-off of 2) and Gene Ontology classifications.
(XLSX)

Table S8 **Protein binding specific genes.** A dataset containing probe sets from the GeneChip Mouse Exon 1.0 ST Array, with raw (normalised) data for E15 and adult mouse, fold change between embryo and adult (with a fold change cut-off of 2) and Gene Ontology classifications.
(XLSX)
