# Correction: Molecular Characterisation of Transport Mechanisms at the Developing Mouse Blood–CSF Interface: A Transcriptome Approach

**DOI:** 10.1371/annotation/2a9099a5-688b-4def-95a7-6ac13b10d096

**Published:** 2012-07-09

**Authors:** Shane A. Liddelow, Sally Temple, Kjeld Møllgård, Renate Gehwolf, Andrea Wagner, Hannelore Bauer, Hans-Christian Bauer, Timothy N. Phoenix, Katarzyna M. Dziegielewska, Norman R. Saunders

An affiliation for the fourth and seventh authors, Renate Gehwolf and Hans-Christian Bauer, was not indicated. Renate Gehwolf and Hans-Christian Bauer are also affiliated with: 7. Paracelsus Medical University, Salzburg, Austria 

