# Supplementary material for: Molecular Characterisation of Transport Mechanisms at the Developing Mouse Blood–CSF Interface: A Transcriptome Approach
Source: PLoS One. 2012 Mar 21;7(3):e33554. doi: 10.1371/journal.pone.0033554 (PMC3310074; doi:10.1371/journal.pone.0033554)
Supplement: Table S1 — MIAME Compliance Checklist. MIAME describes the Minimum Information About a Microarray Experiment that is needed to enable the interpretation of the results of the experiment unambiguously and potentially to reproduce the results [26]. (DOCX) [file pone.0033554.s001.docx]

**Table S1. Total and plasma protein positive choroid plexus epithelial cells during normal mouse development**

| **Age** | ***n*** | **Total Plexus Cells** | **Plasma Protein Positive** | |
| --- | --- | --- | --- | --- |
|  |  |  | **Number of Cells** | **% Total Cells** |
| **E12** | 6 | 0 | 0 | 0 |
| **E13** | 6 | 717 ± 38 | 6 ± 2 | 0.9 ± 0.2 |
| **E14** | 6 | 902 ± 57 | 47 ± 3 | 5.3 ± 0.2 |
| **E15** | 6 | 1647 ± 123 | 144 ± 22 | 8.8 ± 1.4 |
| **E16** | 6 | 2745 ± 205 | 227 ± 44 | 8.1 ± 1.2 |
| **E19** | 6 | 3234 ± 57 | 245 ± 16 | 7.5 ± 0.2 |
| **P2** | 6 | 3524 ± 624 | 252 ± 67 | 7.1 ± 1.0 |
| **P15** | 6 | 4077 ± 135 | 255 ± 30 | 6.3 ± 0.9 |
| **Adult** | 6 | 6499 ± 917 | 227 ± 28 | 3.5 ± 0.2 |
| ***p* value*** |  | < 0.05 | < 0.05 | < 0.05 |

Data are expressed as mean ± s.e.m. rounded to the nearest whole cell. These values are for cells actually counted and represent approximately 10% of all choroid plexus epithelial cells at each age. % Total cells is the percentage of protein positive cells in relation to total plexus cell numbers. The lateral ventricular choroid plexus was not present in E12 embryos, hence no cell count data is available at this age. These data were used to select ages for microarray screening – the highest percentage of protein positive cells was seen at E15, and this age was compared with adult for the remainder of the study. *n* refers to the number of animals. *comparing youngest and oldest ages. Abbreviations: E, embryonic day; P, postnatal days.
