# Supplementary material for: Molecular Characterisation of Transport Mechanisms at the Developing Mouse Blood–CSF Interface: A Transcriptome Approach
Source: PLoS One. 2012 Mar 21;7(3):e33554. doi: 10.1371/journal.pone.0033554 (PMC3310074; doi:10.1371/journal.pone.0033554)
Supplement: Table S2 — Complete data set. The spreadsheet contains a comprehensive list of probe sets from the GeneChip Mouse Exon 1.0 ST Array, with raw (normalised) data for E15 and adult mouse, fold change between embryo and adult (with a fold change cut-off of 2) and Gene Ontology classifications. (DOCX) [file pone.0033554.s002.docx]

Table S2. MIAME Compliance Checklist. MIAME describes the Minimum Information About a Microarray Experiment that is needed to enable the interpretation of the results of the experiment unambiguously and potentially to reproduce the results [26].

| **Experimental Design** | | | |
| --- | --- | --- | --- |
| Type of experiment: | | | Time course analysis of gene expression in two ages of control Swiss Webster mice (embryonic and adult) |
| Experimental factors: | | | time (embryonic day 15 (E15) versus adult) |
| Total number of hybridizations: | | | 3 |
| Type of reference: | | | None |
| Hybridization design: | | | Simple loop pairing with 6 groups (see figure below): |
|  | | | |
| Types of replicates: | | | 3 x biological replicates at each time point (E15 and adult). E15 material from approximately 100 pooled embryos for each array, adult material from at least 5 pooled animals for each array |
| Quality control: | | | Each of the 6 samples (3xE15, 3xadult) consists of multiple pooled biological samples (see above). In addition, a loop hybridization design was employed (see above) |
|  | | | |
| **Array Design** | | | |
| Platform type: | | | Affymetrix |
| Array features and annotations: | | | Mouse Exon 1.0 ST Array  (www.affymetrix.com/products_services/arrays/specific/mouse_exon.affx) |
|  | | | |
| **Samples, Extract Preparation and Labeling Information** | | | |
| Origin of samples | | |  |
|  | Name: | | Swiss Webster mouse (control) |
|  | Provider: | | Taconic  http://www.taconic.com/wmspage.cfm?parm1=16 |
|  | Characteristics | |  |
|  |  | Gender: | E15 (unsexed), adult (females) |

**Table S1 continued.**

| **Samples, Extract Preparation and Labeling Information (continued)** | | | |
| --- | --- | --- | --- |
|  |  | Age: | E15 and adult (over 3 months) |
|  | Samples: | | Both left and right lateral ventricular choroid plexuses |
| Sample manipulations: | | | Choroid plexuses dissected out under RNase-free PBS, pooled and spun to pellet tissue, then snap frozen at -80^o^C |
| RNA extraction protocol: | | | QIAGEN, Rneasy Plus Mini Kit, including QiaShredder columns, according to manufacturers protocol |
| Hybridization protocol: | | | Standard Agilent protocol  Performed at Gen*NY*Sis Centre for Functional Genomic Microarray Core Facility, University at Albany, Albany, New York, USA |
| Sample labeling protocol: | | | Standard Agilent protocol.  Performed at Gen*NY*Sis Centre for Functional Genomic Microarray Core Facility, University at Albany, Albany, New York, USA |
| External controls: | | | None |
|  | | | |
| **Measurement Data and Specifications** | | | |
| Scanning: | | | GeneChip® Scanner 300 7G.  Performed at Gen*NY*Sis Centre for Functional Genomic Microarray Core Facility, University at Albany, Albany, New York, USA |
| Data files: | | | Raw data supplied in *.CEL files, also available in tab-delimited text format |
| Data Analysis: | | |  |
|  | Gene level: | | Raw data was RMA (Robust Multi-Array) normalized (per chip medium), filtered to include the top 80^th^ percentile of genes expressed (all three replicates should meet this criteria) followed by an unpaired t-test (*p* < 0.05, with Benjamini Hochberg false discovery rate correction). This list was then subjected to a fold-change cut off of 2 |
|  | Exon level: | | Raw data was normalized with PLIER (Probe Logarithmic Intensity Error) with a DABG (Detection Above Background) correction (probeset) *p* < 0.05 and 50% of the probesets marked present in at least 50% of the samples in 2 of 2 conditions). This was followed by a splicing ANOVA, *p* < 0.05 with Benjamini Hochberg false discovery rate correction. This list was then filtered for a splicing index cutoff of 0.5 |
